# Heat and Mass Transfer in Shrimp Hot-Air Drying: Experimental Evaluation and Numerical Simulation

**DOI:** 10.3390/foods14030428

**Published:** 2025-01-28

**Authors:** Jhony T. Teleken, Suélen M. Amorim, Sarah S. S. Rodrigues, Thailla W. P. de Souza, João P. Ferreira, Bruno A. M. Carciofi

**Affiliations:** 1 Faculty of Chemical Engineering, Federal University of Southern and Southeastern Pará, Marabá 68505-080, PA, Brazil; jhony.teleken@unifesspa.edu.br (J.T.T.); suelen.amorim@unifesspa.edu.br (S.M.A.); sarah23@unifesspa.edu.br (S.S.S.R.); thaillawenndy@unifesspa.edu.br (T.W.P.d.S.); 2 Graduate Program in Food Engineering, Federal University of Santa Catarina, Florianópolis 88040-900, SC, Brazil; joaoferreira@alimentos.eng.br; 3 Department of Biological and Agricultural Engineering, University of California, Davis, CA 95616, USA

**Keywords:** seafood, food preservation, mathematical modeling, CFD

## Abstract

Shrimp is one of the most popular and widely consumed seafood products worldwide. It is highly perishable due to its high moisture content. Thus, dehydration is commonly used to extend its shelf life, mostly via air drying, leading to a temperature increase, moisture removal, and matrix shrinkage. In this study, a mathematical model was developed to describe the changes in moisture and temperature distribution in shrimp during hot-air drying. The model considered the heat and mass transfer in an irregular-shaped computational domain and was solved using the finite element method. Convective heat and mass transfer coefficients (57.0–62.9 W/m^2^∙K and 0.007–0.008 m/s, respectively) and the moisture effective diffusion coefficient (6.5 × 10^−10^–8.5 × 10^−10^ m^2^/s) were determined experimentally and numerically. The shrimp temperature and moisture numerical solution were validated using a cabinet dryer with a forced air circulation at 60 and 70 °C. The model predictions demonstrated close agreement with the experimental data ($R^{2}\geq$ 0.95 for all conditions) and revealed three distinct drying stages: initial warming up, constant drying rate, and falling drying rate at the end. Initially, the shrimp temperature increased from 25 °C to around 46 °C and 53 °C for the process at 60 °C and 70 °C. Thus, it presented a constant drying rate, around 0.04 kg/kg min at 60 °C and 0.05 kg/kg min at 70 °C. During this stage, the process is controlled by the heat transferred from the surroundings. Subsequently, the internal resistance to mass transfer becomes the dominant factor, leading to a decrease in the drying rate and an increase in temperatures. A numerical analysis indicated that considering the irregular shape of the shrimp provides more realistic moisture and temperature profiles compared to the simplified finite cylinder geometry. Furthermore, a sensitivity analysis was performed using the validated model to assess the impact of the mass and heat transfer parameters and relative humidity inside the cavity on the drying process. The proposed model accurately described the drying, allowing the further evaluation of the quality and safety aspects and optimizing the process.

## 1. Introduction

Shrimp is one of the most consumed seafood in the world and holds great economic importance for the fishing industry of several countries [1,2]. It is a rich source of proteins with essential amino acids, polyunsaturated fatty acids, vitamin B12, astaxanthin, and other micronutrients like calcium and selenium [3]. However, shrimp is highly perishable, deteriorating rapidly after harvesting due to biochemical reactions and microbial growth [4]. Usually, shrimps are stored under low temperatures or dehydrated to extend their shelf life. Although refrigeration and freezing help preserve the shrimp’s freshness, these methods can be costly and require a well-established cold chain to ensure the product quality [5,6]. On the other hand, drying allows shrimp to be stored at room temperature, reduces the transportation costs, and imparts a pleasant and desirable flavor to the dried product [7,8,9,10].

Among the various drying methods found in the literature, sun drying and convective hot-air drying are the two most commonly used techniques for processing shrimp [11,12,13,14]. Although sun drying does not require specialized equipment or technology, it often leads to lower-quality final products due to the uncontrollable weather conditions and extended drying times. In this sense, convective hot-air drying has become increasingly popular for seafood dehydration on an industrial scale [12]. This method offers several advantages, including shorter drying times, ease of operation, simple process implementation and control, and relatively low capital investment requirements [14].

The convective hot-air drying of shrimps involves simultaneous heat and mass transfer, significantly affecting its structure due to shrinkage [15]. The process uses hot, dry air to supply thermal energy, which promotes water evaporation. As the moisture content decreases, the water activity is reduced, which plays a crucial role in the preservation and shelf life of the product. However, the process also deforms the shrimp due to the combined effects of heat exposure and moisture loss. These changes can impact the texture, appearance, and overall quality. Understanding how the processing parameters such as air temperature, relative humidity, and velocity, and food composition, size, and shape affect these phenomena is crucial for designing and improving the drying systems. Mathematical modeling and numerical simulation have been used to describe shrimp processing [16,17]. However, there are few mechanistic models in the literature coupling heat and mass transfer to this end [2,15,18]. Usually, the drying kinetics of the product are described by using thin-layer models, or the shrimp is assumed to be a long cylinder, and the kinetics data are used to estimate the effective diffusion coefficient of the moisture within the shrimp [19,20,21,22]. The cited models provide limited information about the process because they neglect the actual shrimp shape and are often not applicable to other operational conditions. Models assuming the shrimp is a cylinder simplify the mathematical analysis and reduce the computational effort. However, they fail to capture the temperature and moisture distribution dynamics during drying accurately. Actual data demonstrated that, as expected, the thicker parts take much longer to heat up and dehydrate than the tail [8,23]. Using more realistic geometries in the mathematical model formulation can enhance the accuracy, impacting the prediction of the quality attributes.

Niamnuy et al. [15] developed a physics-based model combining heat and moisture transfer in shrinkable and irregular-shaped material to describe the shrimp drying in a jet-spouted bed dryer. They observed that considering the deformation effect, the model predictions improved compared to the model without deformation, although both approaches showed good descriptions of the experimental data. Nguyen et al. [2] estimated moisture-effective diffusion and convective mass transfer coefficients from experimental drying curves using a Bi–Di correlation (Biot and Dincer numbers). These estimated parameters were then used to predict the shrimp’s moisture and temperature profiles in a tunnel dryer. Abedini et al. [18] applied a model based on heat and mass transfer equations considering an irregular shape and non-deformable shrimp geometry to study the drying process in a natural convection–solar hybrid dryer. They evaluated two approaches for determining the moisture diffusion and mass transfer coefficients (constant or moisture-dependent values), showing that variable parameters reduced the deviation between the simulated and experimental data. However, no study has (i) evaluated the effect of the shrimp geometry (irregular vs. cylindrical) on model predictions, (ii) accounted for the temperature- and composition-dependent thermophysical properties of shrimp, or (iii) separately described the heat transfer mechanisms—convective heat transfer and evaporative cooling due to moisture evaporation—in the model formulation, to develop a more robust and extensible model.

To address the identified research gaps, this study investigated the shrimp drying process, developing and validating a mathematical model that considers the product’s irregular shape while coupling the heat and mass transfer mechanisms. The model was validated under different drying conditions to support the parametric analysis, including the effects of the shrimp geometry and air relative humidity. Furthermore, experimental data were used to estimate the heat transfer coefficient, and an optimization method estimated the moisture diffusion within the shrimp and the convective mass transfer coefficient associated with such process.

## 2. Materials and Methods

### 2.1. Shrimp Sample

**Figure 1:**
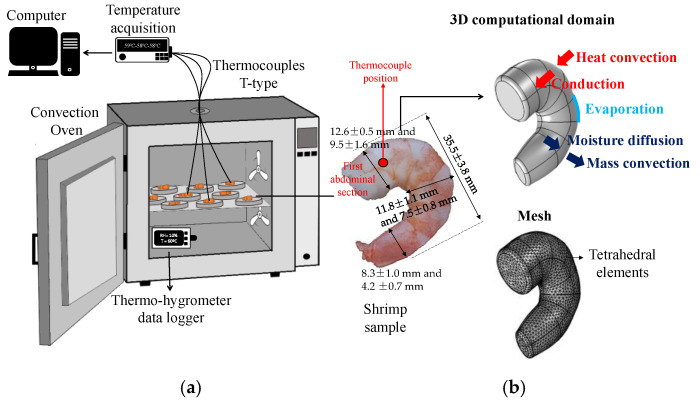
(**a**) Schematic representation of experimental device; (**b**) illustrations of the shrimp sample, computational domain with the heat and mass transport mechanisms considered in the model, and the mesh used in the numerical solution.

Frozen shrimp (*Litopenaeus vannamei*) acquired from the local market (Marabá, Pará, Brazil) were used in this study. The pre-cooked and peeled shrimp were sold in packages with 70 to 110 units per 400 g (CostaSul, Brazil). The individual weight and size of the samples were measured using an analytic scale (resolution 0.0001 g) and digital caliper (resolution 0.01 mm), respectively. The average mass value was 3.94 ± 0.81 g, and the average diameter of the first abdominal segment (Figure 1a) was 11.02 ± 1.95 mm with an average length of 51.6 ± 4.7 mm. The average values represent the measurements obtained using ten shrimps. The gravimetric method determined the moisture content in an oven at 105 °C for 24 h [24]. The pH was measured using a digital Meter (Tecnopon, MPA-210, Piracicaba, SP, Brazil), for which approximately 20 g of shrimp was homogenized with 80 mL of distilled water. The pH measurements were replicated six times.

The moisture content (3.64 ± 0.32 kg/kg on a dry basis; 78.44 ± 1.49% on a wet basis) and pH (6.88 ± 0.02) of the shrimp used in this study agree with the literature values: 75–80% for the moisture content [22,25,26] and pH 6.62–7.04 [27,28,29].

The shrimp computational geometry (Figure 1b) was created in the CAD Tool of COMSOL Multiphysics^TM^, following a visual inspection of the shrimp samples and size measurements that included the length and the semiaxis (bigger diameter and smaller diameter) of the elliptical cross-section of the shrimp in three positions throughout the sample (Figure 1a). The volume of the computational domain was 3.92 cm^3^, and the experimental volume obtained by the fluid displacement method was 3.82 ± 0.76 cm^3^, i.e., an error lower than 3%. A shrimp sample was placed in a graduated cylinder (resolution 0.2 mL) with *n*-heptane, and the displaced volume was determined [30]. This procedure was repeated ten times using different shrimp samples.

### 2.2. Drying Procedure

The shrimp were thawed at room temperature for around 30–40 min (26–28 °C and 40–60% RH). The excess water on the samples’ surface was removed using towel paper, and then each shrimp was placed individually in the aluminum trays (100 mm in diameter, 10 mm in height, and 0.5 mm wall thickness). Next, the samples were placed in a pre-heated convection oven (Nova Ética, model 400-2ND, Vargem Grande Paulista, SP, Brazil) at 60 and 70 °C (Figure 1a). During drying, samples were taken every 15 min for the first 60 min and then every 30 min until 180 min for moisture content measurement. Experimental drying curves (moisture content on a dry basis, $X_{db}$, vs. time, t) were obtained in triplicate for each experimental condition. The shrimp temperature was measured using T-type thermocouples between the shrimp’s first and second abdominal sections (Figure 1a). The thermocouples were connected to a data logger system that recorded measurements at 30 s intervals (Novus, LogBoxBLE, Canoas, RS, Brazil). Inside the dryer cavity, the air temperature ($T_{air}$) and relative humidity (RH) were measured using a digital thermo-hygrometer data logger (UNI-T, model UT330B, Dongguan City, Guangdong Province, China) (Figure 1a).

### 2.3. Mathematical Modeling

A three-dimensional (3-D) model was proposed to describe simultaneous heat and mass transfer in the shrimp during convective drying. Figure 1b illustrates the irregular shape of the computational domain and the transport mechanisms considered in the mathematical model. It was assumed that heat was transferred from the air to the shrimp via convection and within the shrimp via conduction as temperature gradients were developed in all directions. At the same time, the water evaporates at the shrimp’s surface, and the moisture gradients promote water transport by diffusion in the direction of the surface.

The governing equation for water transport within the shrimp is based on the conservation of mass and Fick’s Law for diffusion, as given by Equation (1). Using energy conservation and Fourier’s Law, the heat transfer within the product was described using Equation (2) [31].

(1) $$\frac{\partial c_{w}}{\partial t}=\vec{\nabla }\cdot \left(D\vec{\nabla }c_{w}\right)$$

(2) $$\rho_{sh}c_{p,sh}\frac{\partial T}{\partial t}=\vec{\nabla }\cdot \left(k_{sh}\vec{\nabla }T\right)$$

in which $c_{w}$ is the moisture concentration (kg/m), D is the effective moisture diffusion coefficient of water in the shrimp (m^2^/s), T is the temperature (K), $\rho_{sh}$ is the shrimp density (kg/m^3^), $c_{p,sh}$ is the shrimp specific heat capacity (J/kgK), $k_{sh}$ is the shrimp thermal conductivity (W/mK), and t is the time (s).

The moisture volumetric concentration ($c_{w,0}$) homogeneously distributed in the shrimp was used as the initial condition to solve Equation (1), and the constant and homogeneous temperature ($T_{0}$) was used to solve Equation (2).

(3) $$c_{w}\left(x,y,z,0\right)=c_{w,0}$$

(4) $$T\left(x,y,z,0\right)=T_{0}$$

Equation (5) represents the boundary condition between the shrimp and the air for mass transfer during drying, which describes the amount of water that leaves the sample through evaporation, as proposed in Parisotto et al. [16].

(5) $$\vec{n}\cdot \left(-D\vec{\nabla }c_{w}\right)=h_{m}M_{w}\left[a_{w}\frac{P_{sat,sh}}{RT}-\left(\frac{RH}{100}\right)\frac{P_{sat,air}}{RT_{air}}\right]$$

in which $h_{m}$ is the convective mass transfer coefficient (m/s), $M_{w}$ is the molar mass of water (18 g/mol), $a_{w}$ is the water activity of the shrimp (between 0.0 and 1.0, it was described by a moisture sorption isotherm as a function of the local moisture content on a dry basis, $X_{db}$ (kg/kg)), RH is the relative humidity of the air inside the oven (%), R is the gas constant (8.314 J/mol∙K), $P_{sat}$ is the vapor pressure of water (Pa) estimated with the shrimp surface temperature ($P_{sat,sh}$) or the drying air temperature ($P_{sat,air}$), and $\vec{n}$ is the unit normal vector of the surface.

The heat transfer from the oven air to the product and the heat to evaporate the water on the surface of the shrimp were incorporated into the boundary condition for solving Equation (2), as defined by Equation (6) [16]. Radiation was excluded from the model as it has minimal impact on the heat transfer at oven temperatures up to 70 °C, particularly since the oven walls are made of polished stainless steel. Furthermore, the conductive thermal resistance of the aluminum tray ($R_{cond}=\frac{L_{al}}{k_{al}}=\frac{0.0005\;m}{229\;W/mK}\approx 2.2\times 10^{-6}$ m^2^∙K/W) is much lower than the convective resistance ($R_{conv}=\frac{1}{h_{T}}=\frac{1}{57\;W/m^{2}K}\approx 0.018$ m^2^∙K/W) between the sample and air. Therefore, the effect of the aluminum tray on the heat transfer at the shrimp’s surface is negligible.

(6) $$\vec{n}\cdot \left(-k_{sh}\vec{\nabla }T\right)=h_{T}\left(T-T_{air}\right)-h_{m}M_{w}\left[a_{w}\frac{P_{sat,sh}}{RT}-\left(\frac{RH}{100}\right)\frac{P_{sat,air}}{RT_{air}}\right]∆H_{evap}$$

in which $h_{T}$ is a convection heat transfer coefficient (W/m^2^∙K), and $∆H_{evap}$ is the latent heat of the water vaporization (J/kg).

### 2.4. Model Parameters

#### 2.4.1. Thermophysical Properties of the Shrimp

The shrimp was assumed to be an isotropic and non-deformable medium, and its thermophysical properties are a function of local composition (water and protein content) and temperature. The density ($\rho_{sh}$), specific heat capacity ($c_{p,sh}$), and thermal conductivity ($k_{sh}$) were calculated using Equations (7), (8), and (9), respectively [32].

(7) $$\frac{1}{\rho_{sh}}=\frac{x_{w}}{\rho_{w}}+\frac{x_{pr}}{\rho_{p}}$$

(8) $$c_{p,sh}={x_{w}c}_{p,w}+{x_{pr}c}_{p,p}$$

(9) $$k_{sh}=\frac{1}{2}\left({v_{w}k}_{w}+{v_{pr}k}_{p}\right)+\frac{1/2}{\left({v_{w}/k}_{w}+{v_{p}/k}_{p}\right)}$$

in which $x_{w}=c_{w}/(c_{w}+c_{s})$ is the mass fraction of water (kg/kg) and $x_{p}=1-x_{w}$ is the mass fraction of proteins; $c_{s}$ is the solid volumetric concentration in the shrimp (kg/m^3^); $v_{w}=x_{w}\rho_{sh}/\rho_{w}$ is the volume fraction of water (m^3^/m^3^); and $v_{p}=x_{p}\rho_{sh}/\rho_{p}$ is the volume fraction of proteins. The density, heat capacity, and thermal conductivity of each component as a function of the temperature are summarized in Table 1.

#### 2.4.2. Moisture Isotherm and Water Vapor Pressure

Equation (10) was used to predict shrimps’ water activity ($a_{w}$) based on their moisture content ($X_{db}$). It is a sigmoid function that outputs $a_{w}$ ranging between 0 and 1. To determine the model parameters ($A_{T_{air}}$, $B_{T_{air}}$, and $C_{T_{air}}$), Equation (10) was fitted to the experimental data of the moisture desorption isotherm at 60 °C and 70 °C ($a_{w}$ values between 0.1 and 0.85) obtained by Prachayawarakorn et al. [33]. The relationship between vapor pressure and temperature for water was established using the Antoine equation (Equation (11)) [34].

(10) $$a_{w}=\exp\left[-A_{T_{air}}\exp\left(-B_{T_{air}}X_{db}^{C_{T_{air}}}\;\;\right)\right]$$

(11) $$P_{sat}=10^{3}\exp\left(16.3872-\frac{3885.7}{T+42.83}\right)$$

in which $X_{db}=c_{w}/c_{s}$ is the moisture content on a dry basis (kg/kg), $c_{s}$ is the solid volumetric concentration in the shrimp (kg/m^3^), and $A_{T_{air}}$ ($A_{60}=6.03$, $A_{70}=11.09$), $B_{T_{air}}$ ($B_{60}=11.00$, $B_{70}=11.54$), and $C_{T_{air}}$ ($C_{60}=0.0980$, $C_{70}=0.7195$) are the fitting parameters.

#### 2.4.3. Convective Heat Coefficient Experimental Estimation

**Figure 2:**
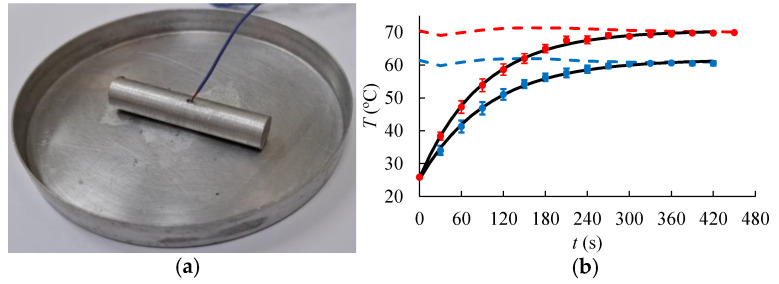
(**a**) Aluminum object used for *h_T_* estimation; (**b**) experimental data of aluminum temperature (points) and the fit of Equation 12 (black line). The dashed lines indicate the experimental air temperature.

The convective heat transfer coefficient ($h_{T}$) was calculated using the lumped capacity method as described in Rodrigues et al. [35]. A cylindrical-shaped object made of aluminum with dimensions similar to the shrimp samples (10.21 mm diameter and 50.68 mm length) was used to perform the experiments. The metallic cylinder was subjected to the same environment used later for the drying essays, i.e., it was placed on an aluminum tray and heated under the same conditions and in the same convective oven used for the shrimp samples (as detailed in Section 2.2), and the temperature was measured using a T-type thermocouple inserted in the center of the object (Figure 2a). The experiments were performed in triplicate for each condition.

It was assumed that the conductive resistance in the aluminum piece is much smaller than the external convective resistance, i.e., the Biot number is lower than 0.1 [36]. Therefore, the thermal energy balance, as presented in Equation (12), describes the heating process of the aluminum object.

(12) $$m_{al}c_{p,al}\frac{dT}{dt}\;\;=h_{T}(T_{air}-T)\;T\left(0\right)=T_{0}$$

in which $m_{al}$ and $A_{al}$ are the mass (11.1214 g) and surface area (0.0017893 m^2^) of the aluminum object, respectively, and $c_{p,al}$ is the heat capacity of the aluminum (900 J/kgK). The solution to Equation (12) leads to Equation (13), which was fitted to the experimental temperature data (see Figure 2b) to determine the heat transfer coefficient.

(13) $$T=T_{air}+(T_{0}-T_{air})\exp\left(-\frac{h_{T}A_{al}}{m_{al}c_{p,al}}t\right)$$

The obtained $h_{T}$ values were 57.0 W/m^2^K at 60 °C, and 62.9 W/m^2^K at 70 °C. The Biot number ($Bi=\frac{h_{T}\;(V_{al}/A_{al})\;}{k_{al}}$, in which $V_{al}$ and $k_{al}$ are the volume, 4.15 × 10^−6^ m^3^, and thermal conductivity, 229 W/mK, of the aluminum, respectively) was calculated to be lower than 6.5 × 10^−4^ for both temperature conditions. These Bi values indicate that the lumped capacitance method is valid [36]. The obtained heat transfer coefficients are in accordance with Ratti and Crapiste [37], who found values ranging from 25 to 95 W/m^2^K during the drying of slices and cylinders of vegetables with an air temperature between 40 and 65 °C and air velocity ranging from 1 to 5 m/s.

#### 2.4.4. Convective Mass Transfer Coefficient and Moisture Effective Diffusion Coefficient

The convective mass transfer ($h_{m}$) and moisture effective diffusion (D) coefficients were determined by fitting the model solution to the experimental data using an exhaustive optimization method [38]. The model was solved by screening $h_{m}$ values from 0.005 to 0.01 m/s at intervals of 0.001 m/s and D from 5 × 10^−10^ to 1 × 10^−9^ m^2^/s at intervals of 0.5 × 10^−10^ m^2^/s [39]. The $h_{m}$ and D values that minimized the root mean squared error (RMSE) between the numerical and experimental temperature data were selected as the optimal values.

### 2.5. Numerical Solution

**Figure 3:**
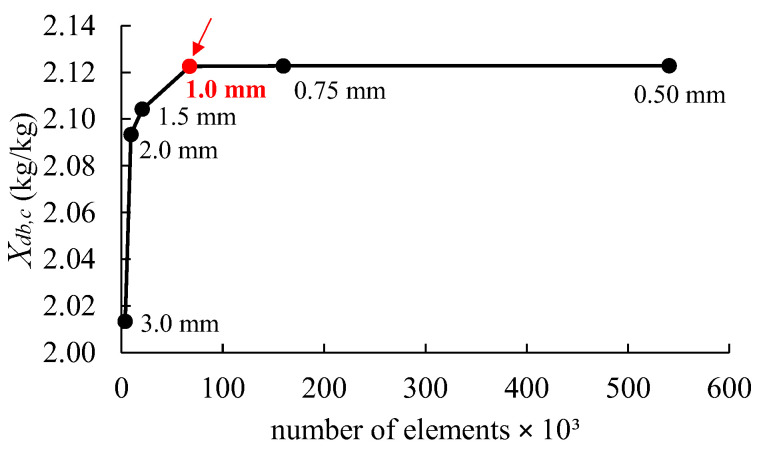
Influences of the number of elements on the calculated moisture content in the center of the shrimp (*X_db_*_,*c*_) during drying at 60 °C.

The mathematical model (Equations (1)–(6)) was solved using the Finite Element Method in the COMSOL Multiphysics^TM^ (version 6.2) software with the Chemical Reaction Engineering and Heat Transfer modules. The computational domains were discretized using first-order tetrahedral elements with a maximum element size of 1 mm (Figure 1b). The direct solver PARDISO was used to solve the system of linear equations, and the time-dependent problem was solved by using the Backward Differentiation Formula (BDF) solver with a maximum time step of 10 s. A study of mesh independence was conducted to determine the ideal mesh size for the model numerical solution. The center moisture content ($X_{db,c}$) of the shrimp after 180 min of drying was used as a criterion for selecting the mesh size [40]. Figure 3 illustrates how the $X_{db,c}$ values change as the elements increase, converging to a specific value. Based on these results, an element size of 1.0 mm, which corresponded to 67,252 elements and 24,822 degrees of freedom, was used for the model numerical solution.

### 2.6. Statistical Parameters

The coefficient of determination ($R^{2}$, Equation (14)) and root mean square error (RMSE, Equation (15)) were used to evaluate the goodness of fit between the model predictions ($Y_{cal,j}$) and the experimental data ($Y_{exp,j}$) for the temperature and moisture.

(14) $$R^{2}=1-\frac{{\sum_{j=1}^{J}\left(Y_{exp,j}-Y_{cal,j}\right)}^{2}}{{\sum_{j=1}^{J}\left(Y_{exp,j}-{\bar{Y}}_{exp}\right)}^{2}}$$

(15) $$RMSE=\sqrt{\frac{{\sum_{j=1}^{J}\left(Y_{exp,j}-Y_{cal,j}\right)}^{2}}{J}}$$

in which J is the total number of experimental data points.

## 3. Results and Discussion

### 3.1. Model Validation

**Figure 4:**
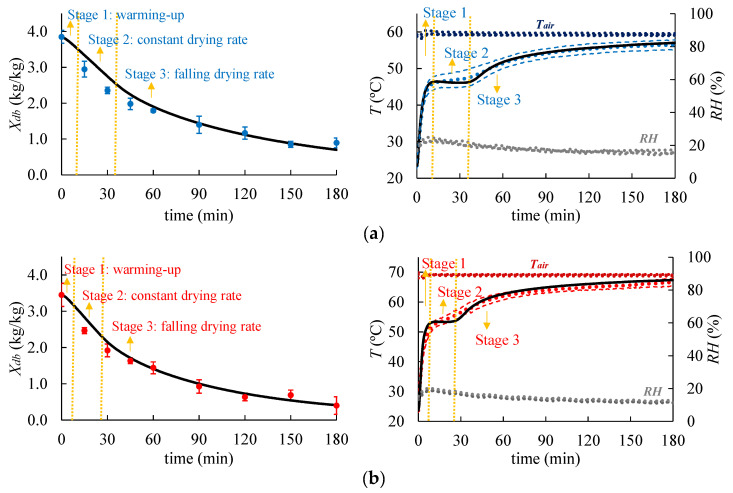
Moisture content (left side) and mid-layer temperature (right side) curves obtained experimentally (dots) and predicted by the mathematical model (continuous lines) during shrimp drying at 60 °C (**a**) and 70 °C (**b**). Values were calculated using a computational domain discretized with a maximum element size of 1 mm.

The comparisons between the calculated and experimental values of the moisture content ($X_{db}$) and temperature (T) of the shrimp during the drying process at 60 and 70 °C are presented in Figure 4. Additionally, it presents the air temperature ($T_{air}$) and relative humidity (RH) inside the dryer cavity. The model predictions demonstrated close agreement with the experimental data. It revealed that the drying process consists of three stages, namely warming up, constant drying rate, and falling drying rate, as detailed in the next section. The statistical parameters $R^{2}$ were greater than 0.95, and the RMSE was lower than 1.12 °C and 0.22 kg/kg, as shown in Table 2, confirming the accuracy of the predictions. For drying at 60 °C, the estimated values for the moisture diffusivity (D) and mass transfer coefficient ($h_{m}$) were 6.5 × 10^−10^ m^2^/s and 0.008 m/s, respectively. At 70 °C, these values were 8.5 × 10^−10^ m^2^/s and 0.007 m/s. The values of the moisture diffusion coefficient are consistent with those reported by Panagiotou et al. [41] for drying fish and fishery products, which ranged between 10^−11^ and 10^−9^ m^2^/s. The estimated $h_{m}$ values are similar to those found by Poós and Varju [42] regarding water evaporation under forced convection at various air temperatures and velocities. The reported values range from 0.007 m/s at 18.5 °C to 0.006 m/s at 60.2 °C, with a constant air velocity of 1 m/s. Additionally, at a temperature of 40 °C, they found values between 0.0029 m/s and 0.0108 m/s as the air velocity increased from 0.27 m/s to 2.02 m/s.

### 3.2. Dynamic of Heat and Mass Transfer in the Shrimp During Drying

**Figure 5:**
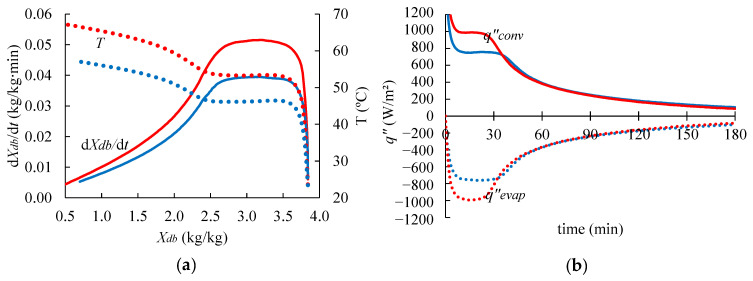
(**a**) Total drying rate (*dX_db_*/*dt*, continuous lines) and temperature (*T*, dotted lines) of the shrimp as a function of the moisture content (*X_db_*) over drying at 60 °C (blue) and 70 °C (red). (**b**) Average heat flux transferred to the shrimp via convection ($q_{conv}^{\prime \prime }\;$, continuous lines) and evaporative heat flux ($q_{evap}^{\prime \prime }$, dotted lines) in the shrimp surface over-drying at 60 °C (blue) and 70 °C (red). Numerical results used computational domain discretized with a maximum element size of 1 mm.

The shrimp drying curves (Figure 5) present three distinct stages (warming up, constant drying rate, and falling drying rate), which are often observed in the convective drying process of solid foods [43]. Initially, there is a brief period of rapid heating and a rising drying rate ($dX_{db}/dt$). Next, there was observed a period in which the temperature remained mostly constant (around 46 °C and 53 °C for the process at 60 °C and 70 °C, respectively) and the drying rate reached its maximum value (Figure 5a). During this stage, the heat is transferred from the surroundings to the surface of the shrimp through convection (estimated by reference to the convective heat flux, $q_{conv}^{\prime \prime }$, W/m^2^) and used in the water evaporation (described by reference to the evaporative heat flux, $q_{evap}^{\prime \prime }$, W/m^2^). The latent heat of evaporation prevented a temperature rise since they are very close to each other, i.e., $q_{conv}^{\prime \prime }\approx q_{evap}^{\prime \prime }$, as shown in Figure 5b. Subsequently, the temperature rises again, approaching the air temperature, and the drying rate gradually decreases. In this stage, the drying process is governed by the mass transfer within the shrimp.

Figure 5a shows the calculated values of the temperature (T) and the drying rate ($dX_{db}/dt$) of shrimp as a function of the moisture content ($X_{db}$). As illustrated, the maximum drying rate for the process at 70 °C (0.05 kg/kg∙min) was higher than at 60 °C (0.04 kg/kg∙min). Moreover, the constant drying rate period was shorter for the process at 70 °C than at 60 °C. Delfiya et al. [44] found around 0.025 kg/kg∙min using hot air at 45 °C and 1.5 m/s for drying shrimp. These results were expected since a higher temperature leads to a higher heat flux to the shrimp (Figure 5b), resulting in a higher moisture flux from the shrimp to the air [45]. Also, it was demonstrated that the higher the drying temperature, the higher the equilibrium moisture on the shrimp’s surface ($a_{w}\frac{P_{sat,sh}}{RT}$) and the higher the moisture diffusion coefficient (see Section 3.1). Additionally, the relative humidity (RH) in the dryer working at 60 °C ranged between 16 and 23%, while the dryer working at 70 °C ranged between 11 and 19% (as shown in Figure 4), which also increased the rate of moisture loss by the shrimp samples. After 180 min of drying, the shrimp moisture content on a dry basis was 0.69 kg/kg (40.8% on a wet basis) at 60 °C and 0.40 kg/kg (28.6% on a wet basis) at 70 °C.

**Figure 6:**
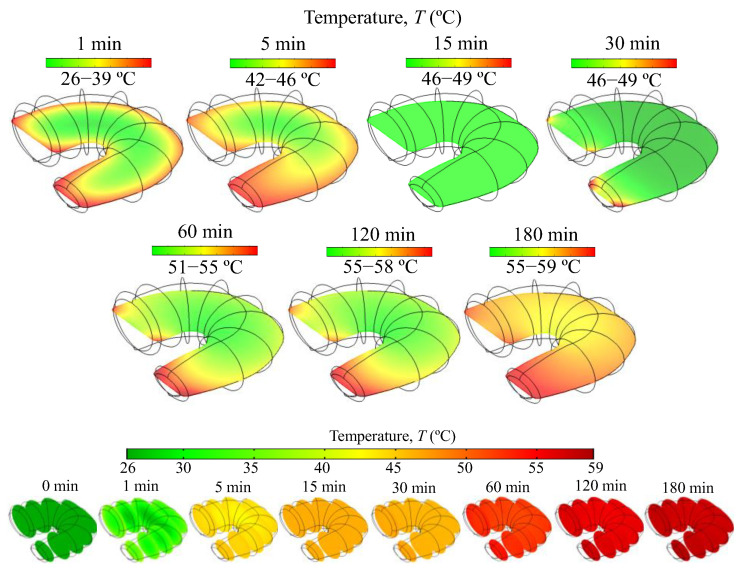
Temperature profiles (*T*) in the mid-layer of the shrimp during the drying at 60 °C calculated using a computational domain discretized with a maximum element size of 1 mm.

**Figure 7:**
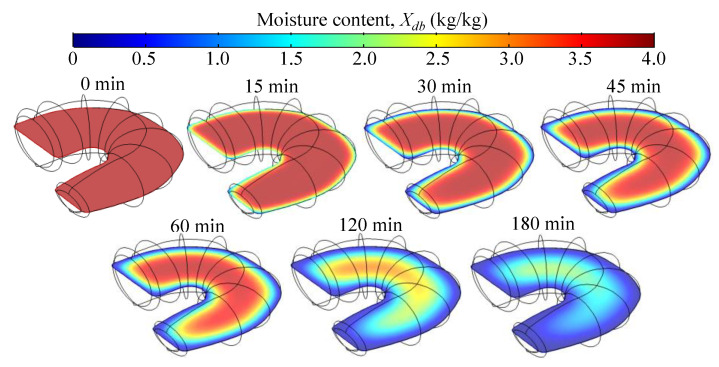
Moisture content profiles (*X_db_*) in mid-layer of the shrimp during the drying at 60 °C calculated using a computational domain discretized with a maximum element size of 1 mm.

Figure 6 and Figure 7 show the spatial distribution of the temperature and moisture within the sample during drying at 60 °C. Initially, the temperature of the shrimp rises rapidly, reaching up to 39 °C after 1 min. Subsequently, after 15 and 30 min, it stabilizes at around 46 °C before gradually increasing after 60, 120, and 180 min, eventually approaching the air temperature of 60 °C (Figure 6). The shrimp’s tail, thinner than the body, experiences a faster temperature increase and moisture loss. According to Figure 7, after 180 min, the moisture content in the shrimp tail and on its surface had decreased to less than 0.2 kg/kg (16.7% on a wet basis), while the moisture content in the inner part of the sample was approximately 1.9 kg/kg (65.5% on a wet basis). During storage, the moisture gradients within the product diminish as moisture continues to diffuse, while water loss nearly ceases, maintaining a constant average moisture content. The predicted moisture profile in shrimp (Figure 7) follows the qualitative behavior observed using magnetic resonance imaging (MRI) to assess the dynamics of moisture change in shrimps during the hot-air drying process [8,23].

### 3.3. Effect of the Shrimp Geometry

**Figure 8:**
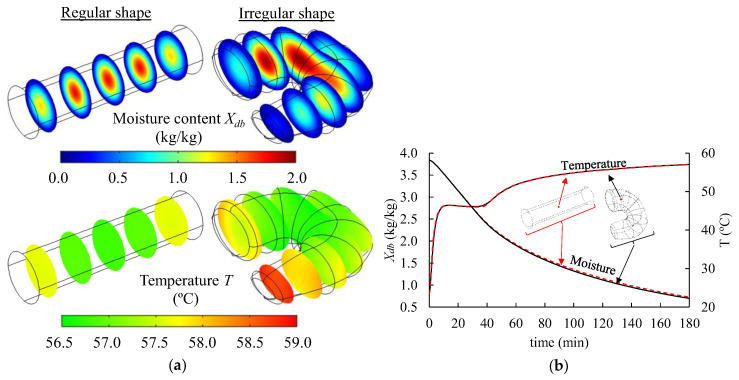
(**a**) Numerical solution for moisture and temperature distribution in the shrimp after 180 min of drying at 60 °C for cylindrical-shaped and irregular-shaped computational domains. (**b**) Moisture content and mid-layer temperature curves (black lines—irregular shape, red lines—cylindrical shape). Values were obtained with computational domain discretized with a maximum element size of 1 mm.

A numerical analysis was performed to evaluate how the selected geometry to represent the shrimp shape impacts the mathematical modeling of the drying process. The shrimp was represented as a finite cylinder (a common assumption in the literature) with dimensions similar to the shrimp samples (11 mm diameter and 42 mm length). The irregular-shaped computational domain had the same volume as the one with a simplified shape. The results differed for both geometries’ moisture content and temperature profiles (Figure 8a). However, the moisture loss curve and temperature at the cold spot were very close (Figure 8b). Therefore, using a finite cylinder may serve as a simplification for process assessment. Still, a geometry with an irregular shape should be the preferred choice as it offers more realistic information about the moisture and temperature variation inside the shrimp, allowing a much more accurate description of the quality parameters that differentiate what happens in thinner and thicker regions. Moreover, regarding the numerical solution of the models, the number of degrees of freedom was 24,822 for the irregular shape and 23,670 for the cylindrical shape, resulting in similar computation times, which also do not justify the use of a simplified geometry.

### 3.4. Parametric Sensitivity Analysis

**Figure 9:**
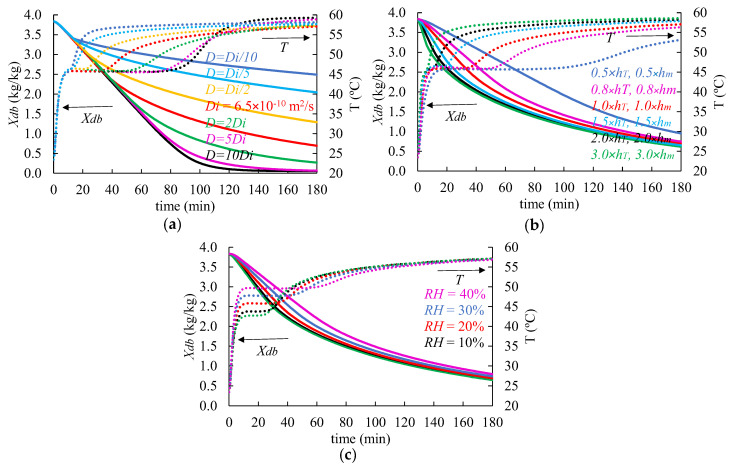
Temperature (*T*) and moisture (*X_db_*) kinetics resulting from parametric analysis of (**a**) diffusion coefficient (*D*), (**b**) convective transfer coefficients (*h_T_* and *h_m_*), and (**c**) relative humidity of air (*RH*) during shrimp drying at 60 °C. Numerical results obtained using a computational domain with a maximum element size of 1 mm.

Based on the validated model, numerical simulations were conducted to evaluate the influence of the moisture diffusion coefficient (D) and convective transfer coefficients ($h_{T}$ and $h_{m}$) on the shrimp drying process. The simulations were performed for the shrimp drying at 60 °C. Figure 9a shows that a higher D value, i.e., a lower inner resistance of mass transfer, resulted in a faster decrease in moisture and an extension of the period of the constant drying rate. Defraeye and Verboven [46] observed the same effect of D on the dynamics of change in the temperature and moisture content in apples during the convective drying process. Unlike the diffusion coefficient D, which is an intrinsic property of the food, the values of $h_{T}$ and $h_{m}$ are influenced by the process conditions, particularly the airflow within the dryer, sample geometry, the thermophysical properties of the air, and the flow regime (whether laminar or turbulent) [36]. Specifically, as the air velocity increases, these coefficients rise due to a reduction in the thickness of the boundary layers around the shrimp or changes in the flow regime, resulting in shorter drying times, as shown in Figure 9b. However, up to a certain point (around 2.0 × $h_{T}$ = 114 W m^−2^K^−1^ and 2 × $h_{m}$ = 0.016 m s^−1^), further increases in the convective transfer coefficients have a diminishing effect on the drying time (internal resistance to mass transfer becomes much more relevant). The time necessary to reduce the moisture content of shrimp (to 1 kg kg^−1^) was 174, 142, 133, 124, 120, and 117 min for 0.5, 0.8, 1.0, 1.5, 2.0, and 3.0 × $h_{T}$ and $h_{m}$, respectively. In such conditions, higher temperatures and the lower relative humidity of the air were more effective in removing the remaining moisture from the food than increasing the air velocity in the cavity.

Figure 9c illustrates how the relative humidity of air (RH) impacts the drying. This process parameter influences the driving force of moisture transfer by altering the difference $\left[a_{w}\frac{P_{sat,sh}}{RT}-\left(\frac{RH}{100}\right)\frac{P_{sat,air}}{RT_{air}}\right]$, which affects the evaporative flow at the shrimp surface. It explains the lower drying rate and higher temperatures of the shrimp dried at 40% of RH (0.03 kg kg^−1^min^−1^ and 49.6 °C during the period of the constant drying rate) compared to those processes at 5% of RH (0.05 kg kg^−1^min^−1^ and 42.7 °C). When the shrimp’s surface has no more moisture, the internal resistance to mass transfer becomes the limiting factor for the drying. This was observed in the present study when evaluating the drying curves after 120 min. There, the difference in the drying rate between 40% (0.0086 kg kg^−1^min^−1^) and 5% of relative humidity (0.0101 kg kg^−1^min^−1^) was around only 15%. By controlling the RH in the oven, the drying time needed to reach a shrimp moisture content of 1 kg kg^−1^ was reduced from 140 min (40% RH) to 125 min (5% RH). The effect of this process parameter on the shrimp’s moisture content and temperature curve is consistent with the literature data on the convective drying of food products [40,47].

## 4. Conclusions

The three-dimensional mathematical model based on heat and mass transfer phenomena successfully simulated shrimp dehydration in a cabinet dryer with forced air circulation, as validated by the excellent agreement with the experimental data on the moisture content and temperature (R^2^ > 0.95). The model demonstrated how the temperature and moisture changed over time within the shrimp during drying. It also identified three distinct stages in the drying kinetics: the warming up, the constant drying rate, and the falling drying rate phases. The irregular shape of the computational domain did not require additional numerical effort compared to the simplified cylindrical shape. However, the irregular-shaped domain showed the possibility of differentiating the shrimp body parts over time, allowing a further quality assessment. Specifically, the moisture content at the shrimp tail was significantly lower than at its center, while the temperature differences were relatively small. The isolated evaluation of the convective heat transfer and evaporative cooling is another advantage of this model, as it enables predicting shrimp drying under varying process conditions (e.g., relative humidity, air velocity, and temperature). Furthermore, the convective heat and mass coefficients and moisture effective diffusion coefficient experimental and numerical procedures were valuable in estimating the parameters. Overall, the proposed model is a powerful tool that can be used for controlling and optimizing the drying process and for predicting the moisture content and temperature under various drying conditions.

## Figures and Tables

**Table 1:** Equations for predicting the thermophysical properties of water and proteins [32].

Property	Equation ^1^
Density (kg/m^3^)	
$\rho_{w}$	$997.18+3.1439\times 10^{-3}T-3.7574\times 10^{-3}T^{2}$
$\rho_{p}$	1329.9−0.51840T
Heat capacity (J/kgK)	
$c_{p,w}$	$4176.2-0.0909T+5.4731\times 10^{-3}T^{2}$
$c_{p,p}$	$2008.2+1.2089T-1.3129\times 10^{-3}T^{2}$
Thermal conductivity (W/mK)	
$k_{w}$	$0.57109+1.762\times 10^{-3}T-6.7036\times 10^{-6}T^{2}$
$k_{p}$	$0.17881+1.958\times 10^{-3}T-2.7178\times 10^{-6}T^{2}$

^1^ Temperature, *T*, in Celsius degree.

**Table 2:** *R*^2^ and *RMSE* obtained by comparing experimental and predicted values of temperature and moisture content on a dry basis during the drying of shrimp at 60 and 70 °C.

Temperature (°C)	$R_{T}^{2}$	$R_{X_{db}}^{2}$	$RMSE_{T}$ (°C)	$RMSE_{X_{db}}$ (kg/kg)
60	0.99	0.95	0.50	0.22
70	0.97	0.97	1.12	0.16

## Data Availability

The original contributions presented in the study are included in the article; further inquiries can be directed to the corresponding author.
